# Longitudinal Assessment of Cardiac Native T1 in Transfusion-Dependent Thalassemia

**DOI:** 10.31083/RCM50439

**Published:** 2026-06-29

**Authors:** Antonella Meloni, Laura Pistoia, Vincenzo Positano, Ilaria Fotzi, Antonella Cossu, Emanuela De Marco, Elisabetta Corigliano, Cristina Paci, Alberto Clemente, Andrea Barison

**Affiliations:** ^1^Bioengineering Unit, Fondazione G. Monasterio CNR-Regione Toscana, 56124 Pisa, Italy; ^2^Unità Operativa Complessa Ricerca Clinica, Fondazione G. Monasterio CNR-Regione Toscana, 56124 Pisa, Italy; ^3^SOC Oncologia, Ematologia e Trapianto di Cellule Staminali Emopoietiche, Meyer Children’s Hospital IRCCS, 50139 Firenze, Italy; ^4^Ambulatorio Trasfusionale - Servizio Immunoematologia e Medicina Trasfusionale Dipartimento dei Servizi, Presidio Ospedaliero “San Francesco” ASL Nuoro, 08100 Nuoro, Italy; ^5^UO Oncoematologia Pediatrica, Azienda Ospedaliero Universitaria Pisana – Stabilimento S. Chiara, 56126 Pisa, Italy; ^6^Ematologia Microcitemia, Ospedale San Giovanni di Dio – ASP Crotone, 88900 Crotone, Italy; ^7^Centro Trasfusionale, Ospedale “S Maria alla Gruccia”, 52025 Montevarchi, Italy; ^8^Department of Radiology, Fondazione G. Monasterio CNR-Regione Toscana, 56124 Pisa, Italy; ^9^Cardiology and Cardiovascular Medicine, Fondazione G. Monasterio CNR-Regione Toscana, 56124 Pisa, Italy

**Keywords:** thalassemia, iron overload, magnetic resonance imaging, left ventricle

## Abstract

**Background:**

Cardiovascular magnetic resonance (CMR) T2* is the reference standard for assessing myocardial iron overload (MIO). Native T1 mapping has emerged as a complementary technique and may be more sensitive for detecting mild or early myocardial iron deposition. We evaluated longitudinal changes in native left ventricular (LV) T1 values over 18 months in patients with transfusion-dependent thalassemia (TDT).

**Methods:**

A total of 64 TDT patients consecutively enrolled in the Extension-Myocardial Iron Overload in Thalassemia (E-MIOT) project underwent two CMR examinations at 1.5T. Native T1 mapping and T2* relaxometry were performed using standardized protocols. LV T1 and T2* values were calculated from 16 myocardial segments. LV ejection fraction was assessed by cine imaging.

**Results:**

At baseline, mean LV T1 was 959.51 ± 101.46 ms and mean LV T2* was 37.17 ± 9.44 ms, with a significant correlation between the two parameters (R = 0.533; *p* < 0.0001). Both LV T1 and T2* were reduced in 9 (14.1%) patients. Meanwhile, 17 (26.6%) patients exhibited reduced LV T1 despite normal LV T2*, whereas only one (1.6%) patient had normal LV T1 in the presence of pathological T2*. Increased LV T1 was observed in two (3.1%) patients. During follow-up, global LV T1 did not change significantly (mean change 1.99 ± 63.57 ms; *p *= 0.841), whereas LV T2* increased significantly (mean change 1.61 ± 4.52 ms; *p* = 0.001). Individual T1 trajectories varied: 22.2% of patients with normal baseline T1 developed reduced T1, while 26.9% of those with reduced baseline T1 normalized at follow-up. Changes in LV T1 were inversely associated with baseline T1 values (R = −0.406,* p* = 0.001) and correlated with changes in T2* (R = 0.311, *p *= 0.012), but not with age, ferritin, hemoglobin levels, or LV ejection fraction.

**Conclusions:**

In well-managed TDT patients, native myocardial T1 values remain overall stable over mid-term follow-up despite marked interindividual variability. Baseline T1 values and parallel changes in T2* influence longitudinal changes in T1, supporting native T1 mapping as a complementary, but not interchangeable, tool to T2* for the assessment and longitudinal monitoring of MIO.

## 1. Introduction

Transfusion-dependent thalassemia (TDT) is a severe hereditary blood disorder caused by defective hemoglobin production, leading to chronic anemia that requires lifelong transfusion therapy from early childhood [1,2,3]. While transfusions are life-saving, they lead to systemic iron accumulation, which can affect the heart and cause significant morbidity and mortality if not properly managed [2,4]. This underscores the importance of accurate assessment and monitoring of myocardial iron deposition. Cardiovascular magnetic resonance (CMR) T2* imaging is widely recognized as the gold standard for quantifying myocardial iron overload (MIO) in TDT patients [5,6,7]. Its routine application has significantly improved clinical management by enabling early identification of patients at higher risk of cardiac dysfunction [8,9] and by guiding the intensification of iron chelation therapy when needed [10,11]. Over the past several years, these strategies have contributed to a progressive reduction in myocardial iron accumulation and associated cardiac complications, with a substantial positive impact on patient survival [12,13,14].

However, T2* measurements can be affected by susceptibility artifacts and other technical limitations [15], which may reduce their sensitivity for detecting mild or early-stage myocardial iron deposition. In this context, native T1 mapping has emerged as a valuable complementary tool. Myocardial iron accumulation not only lowers T2* values but also shortens T1 relaxation times [16], and a significant correlation between the two relaxation times has been reported in patients with TDT and other hemoglobinopathies [17,18,19,20,21,22]. Evidence suggests that T1 mapping can detect iron even in patients whose T2* values fall within the borderline range, highlighting its ability to identify subtle or early iron deposition that might be overlooked by conventional T2* measurements [20,21]. Importantly, in cohorts of well-managed patients with generally mild myocardial iron load, T1 values have been shown to be a more sensitive marker of cardiac involvement, including heart failure and arrhythmias, than T2* measurements [21]. Clinically, reduced T1 may serve as an early indicator of myocardial disease. By preceding changes in T2*, it can reflect progression from simple iron accumulation to subclinical myocardial dysfunction. Early detection of these changes through T1 mapping can help identify patients at higher risk of cardiac complications and guide timely optimization of chelation therapy.

Despite the growing evidence supporting the role of T1 mapping in TDT, all studies have assessed T1 at a single time point, leaving a significant gap in the understanding of intra-individual temporal dynamics. The present study was specifically designed to bridge this gap by providing the first longitudinal evaluation of native myocardial T1 in this population. Accordingly, we evaluated changes in myocardial T1 values between two CMR examinations, with the additional aim of determining whether T1 remains stable at the population level while exhibiting clinically relevant variability at the individual level. By addressing this aspect, we sought to better define the potential role of T1 mapping in the longitudinal monitoring of early cardiac involvement and in supporting individualized risk stratification over time.

## 2. Materials and Methods

### 2.1 Study Population

The Extension-Myocardial Iron Overload in Thalassemia (E-MIOT) project is an Italian multicenter network comprising 66 thalassemia centers and 15 magnetic resonance imaging (MRI) sites that use optimized and standardized MRI protocols and post-processing methods. The participating centers are linked via a dedicated web-based platform that systematically collects demographic, clinical, laboratory, and CMR data.

The E-MIOT project enrolls patients of both sexes and all ages affected by thalassemia syndromes or structural hemoglobin variants. Eligibility criteria included the clinical indication for MRI to assess cardiac and hepatic iron overload, provision of written informed consent, authorization for the use and disclosure of protected health information, and the absence of absolute contraindications to MRI.

**Fig. 1.**
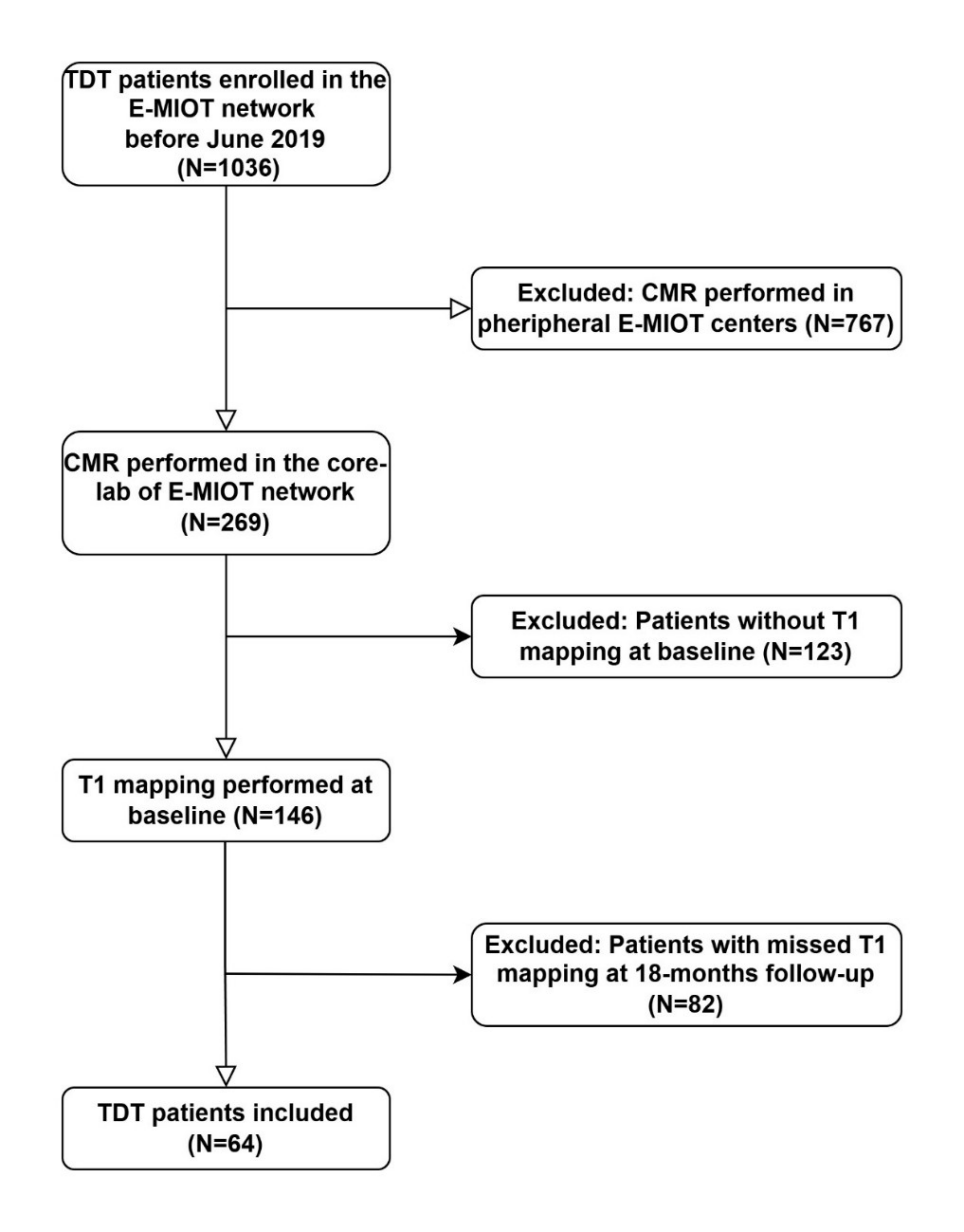
**Flowchart of study enrollment and patient selection**. Of the initial 1036 TDT patients enrolled in the E-MIOT Network before June 2019, 767 were excluded for having CMR performed in peripheral centers rather than the core laboratory (Pisa). Among the remaining 269 patients, 123 lacked baseline T1 mapping data. A further 82 patients were excluded due to a missed 18-month follow-up T1 mapping. The final longitudinal analysis included a cohort of 64 patients. TDT, transfusion-dependent thalassemia; E-MIOT, Extension-Myocardial Iron Overload in Thalassemia; CMR, cardiovascular magnetic resonance; N, number.

We retrospectively included in this study all TDT patients enrolled in the E-MIOT project who underwent two CMR scans—baseline and follow-up—at the reference MRI center in Pisa (Fig. 1). The baseline was defined as the first assessment of myocardial T1 mapping, while the follow-up CMR was performed after an interval of 18 ± 3 months, in accordance with the study protocol.

The study adhered to the principles of the Declaration of Helsinki and was approved by the local ethics committees. Each participant gave written informed consent.

### 2.2 CMR Protocol

CMR examinations were conducted using a 1.5-T scanner (Signa Artist, GE Healthcare, Milwaukee, WI, USA). Image acquisition was performed with a 30-channel cardiac phased-array surface coil under breath-hold conditions and electrocardiogram gating.

Three parallel left ventricular (LV) short-axis slices (basal, medial, and apical) were acquired in end-diastole by a Modified Look-Locker Inversion recovery sequence [3(3s)3(3s)5 scheme] for T1 mapping [23] and a multi-echo gradient echo sequence (10 echo times; echo spacing = 2.26 ms) for T2* relaxometry [24].

Native T1 images were transferred to cvi42 software (Version 5.11.4, Circle Cardiovascular Imaging Inc., Calgary, Alberta, Canada) for offline post-processing [23]. Pixel-wise T1 maps were generated, and endocardial and epicardial borders were manually traced on each short-axis slice, excluding the blood pool and epicardial fat. After identification of the anterior and inferior right ventricular insertion points, the LV was divided into 16 segments [25]. Segmental T1 values were calculated as the mean of all pixels within each segment. The reproducibility of LV T1 mapping was previously established at our center, demonstrating high intra- and inter-observer agreement (coefficient of variability <5%) in both healthy volunteers [23] and TDT patients [21].

T2* images were processed using a validated dedicated software tool (HIPPO MIOT®), as previously described [24]. T2* values were obtained in all 16 myocardial segments using a region-based approach, and a correction map was applied to compensate for susceptibility artifacts [26].

LV T1 and T2* values were obtained by averaging all segmental T1 and T2* values, respectively.

At both time points, previously established site-specific reference ranges were used for T1 and T2* values. Lower and upper limits of normal for LV T1 were 928–1056 ms for males and 983–1091 ms for females [23]. The lower limit of normal for LV T2* was 32 ms.

Steady-state free precession cine images were obtained in contiguous 8-mm short-axis slices spanning from the atrioventricular ring to the apex for evaluation of left ventricular volumes and ejection fraction using a standard approach [27].

### 2.3 Laboratory Assessment

At each participating thalassemia center, biochemical tests were conducted using standardized clinical chemistry systems, following established laboratory procedures. Hemoglobin and ferritin levels were measured at least six times per year for each patient, and the mean values were used in the analyses.

### 2.4 Statistical Analysis

All statistical analyses were performed using SPSS version 27.0 (IBM Corp., Armonk, NY, USA) statistical package.

Continuous data were presented as mean ± standard deviation (SD), whereas categorical variables were reported as absolute counts and percentages.

Data normality was checked using the Kolmogorov-Smirnov test.

Either Pearson’s or Spearman’s tests were utilized for correlation analysis, depending on the nature of the data.

Significant differences between baseline and follow-up measurements were assessed using the paired Student *t*-test for normally distributed variables or the Wilcoxon test for non-normal data.

For continuous variables, the change was calculated as the difference between values at follow-up and baseline MR scans.

Between-groups comparisons for continuous variables were conducted using the independent-samples *t*-test for normally distributed data and the Wilcoxon rank-sum test for non-normally distributed data. For categorical variables, differences between groups were assessed using the chi-squared test or Fisher’s exact test, as appropriate.

A two-tailed *p *< 0.05 was considered statistically significant.

## 3. Results

### 3.1 Baseline Characteristics

Table 1 summarizes the demographic and clinical characteristics of the 64 TDT patients included in this study at the baseline CMR. All patients were white, regularly transfused since early childhood, and chelated. The mean age at the baseline CMR examination was 38.57 ± 11.17 years, and 38 (59.4%) patients were female.

**Table 1.** **Baseline demographic, clinical, and CMR data of the patients**.

Variable		Value
Sex (males/females)		26/38
Age (years)		38.57 ± 11.17
Transfusion starting age (months)		19.29 ± 15.67
Chelation starting age (years)		4.31 ± 6.27
Splenectomy, N (%)		24 (37.5)
Pre-transfusion hemoglobin (g/dL)		9.59 ± 0.44
Serum ferritin (ng/mL)		1259.17 ± 1253.63
LV T1 values (ms)		959.51 ± 101.46
LV T1 categories, N (%)		
	Normal	36 (56.3)
	Reduced	26 (40.6)
	Increased	2 (3.1)
LV T2* values (ms)		37.17 ± 9.44
Reduced LV T2*, N (%)		10 (15.6)
LV end-diastolic volume index (mL/m^2^)		82.97 ± 14.24
LV end-systolic volume index (mL/m^2^)		30.81 ± 8.39
LV stroke volume index (mL/m^2^)		52.16 ± 10.19
LV ejection fraction (%)		63.06 ± 7.01

N, number; LV, left ventricular; CMR, cardiovascular magnetic resonance.

Fifty-five patients (85.9%) had previously undergone at least one T2* CMR scan, whereas myocardial T1 mapping was performed for the first time in all patients.

At baseline, the mean LV T1 value was 959.51 ± 101.46 ms (range: 494.19–1169.75 ms), and the mean LV T2* value was 37.17 ± 9.44 ms (range: 5.19–47.50 ms). A significant correlation was observed between the two relaxation times (R = 0.533;* p *< 0.0001).

Thirty-five (54.7%) patients exhibited normal LV T1 and T2* values. An increased LV T1 value was observed in two (3.1%) patients, all of whom had normal LV T2* values. Only one (1.6%) patient had a normal LV T1 value in the presence of a pathological T2* value. Reduced values of both relaxation times were found in 9 (14.1%) patients. Finally, 17 (26.6%) patients were found to have reduced LV T1 values in the presence of normal LV T2* measurements.

Compared with the rest of the cohort, patients with reduced T1 despite normal T2* were significantly older (44.25 ± 6.09 vs. 36.52 ± 11.90 years; *p* = 0.006), while ferritin levels, chelation regimen, and cardiac function were comparable between groups (all *p* > 0.070).

### 3.2 Changes in CMR Parameters

Mean follow-up time was 18.69 ± 1.97 months (median 18.78 months).

At the follow-up, the mean LV T1 value was 961.50 ± 76.99 ms (range: 706.01–1091.10 ms), and the mean LV T2* value was 38.78 ± 8.91 ms (range: 8.05–51.12 ms). A significant correlation was observed between the two relaxation times (R = 0.494; *p* < 0.0001).

**Fig. 2.**
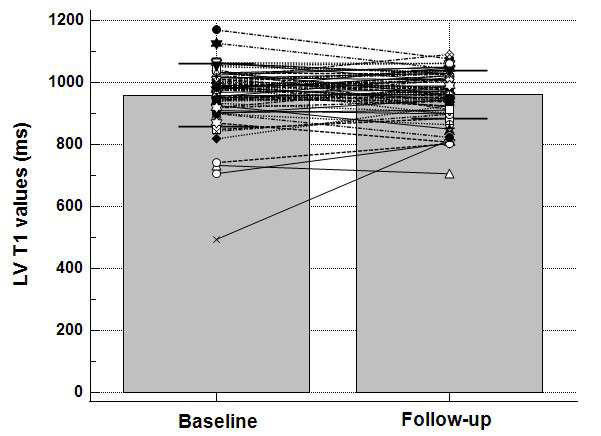
**Longitudinal evolution of LV T1 values**. Individual lines represent patient-specific trajectories, illustrating the direction of change from baseline to follow-up. Shaded bars indicate the mean LV T1 values at each time point, with error bars representing the standard deviation (SD). LV, left ventricular.

Native LV T1 did not significantly change between baseline and follow-up CMR scans (mean difference: 1.99 ± 63.57 ms; *p *= 0.841). Among the 36 patients with a normal baseline LV T1 value, 8 (22.2%) showed a reduced T1 and one (2.8%) an increased T1 at the follow-up. Out of the 26 patients with a reduced LV T1 at the baseline, only 7 (26.9%) showed a normal T1 at the follow-up CMR. All these 7 patients also had a normal LV T2* at the follow-up. Both patients with an increased LV T1 at baseline had a normal T1 at follow-up. Fig. 2 depicts individual patient trajectories of LV T1 values from baseline to follow-up, together with the mean values for the cohort.

At the follow-up CMR, a significant increase in LV T2* values was detected (mean difference: 1.61 ± 4.52 ms; *p *= 0.001). Among the 54 patients with a normal baseline LV T2* value, only one (1.9%) showed a reduced T2* at the follow-up. This patient had a reduced LV T1 at both CMR scans. Out of the 10 patients with a reduced LV T2* at the baseline, only 2 (20%) showed a normal T2* at the follow-up.

No statistically significant changes were detected in LV ejection fraction (mean difference: –0.44 ± 6.54%; *p *= 0.594).

### 3.3 Correlates of Changes in LV T1 Values

Changes in LV T1 values between the two CMR scans were independent of age (R = 0.103; *p *= 0.419) and baseline serum levels of pre-transfusion hemoglobin (R = 0.103; *p *= 0.419) and ferritin (R = 0.187; *p *= 0.322).

**Fig. 3.**
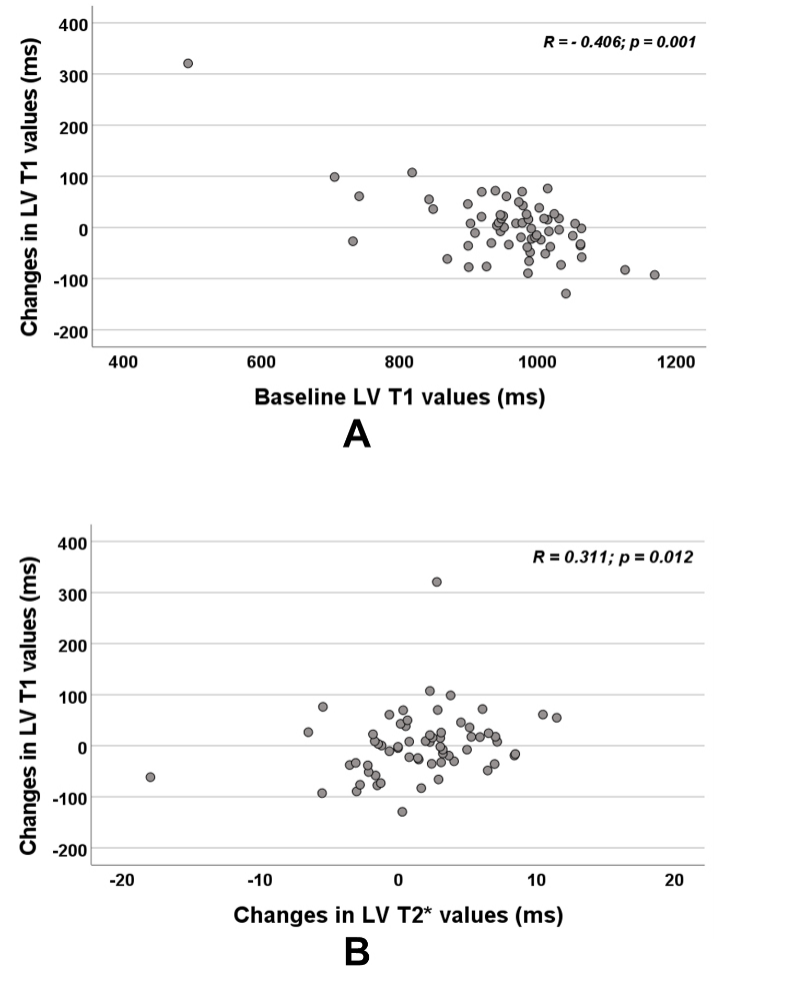
**Correlates of changes in LV T1 values**. (A) Scatter plot of changes in LV T1 values versus baseline LV T1 values. (B) Scatter plot of changes in LV T1 values versus changes in LV T2* values. LV, left ventricular.

A significant inverse association was detected between changes in LV T1 and baseline LV T1 values (R = –0.406;* p *= 0.001) (Fig. 3A). Changes in LV T1 values were significantly different between patients with normal and reduced baseline LV T1 values (–10.09 ± 42.51 vs. 25.64 ± 78.84 ms; *p *= 0.043).

A significant correlation was found between changes in LV T1 and T2* values (R = 0.311; *p* = 0.012) (Fig. 3B), while changes in LV T1 values were not correlated with baseline LV T2* values (R = –0.153; *p* = 0.226).

No association was found between changes in LV ejection fraction and changes in LV T1 (R = –0.109; *p* = 0.390) or LV T2* (R = –0.091;* p *= 0.477) values.

### 3.4 Chelation Therapy

At baseline, deferasirox was the most frequently prescribed chelation therapy, used by 56.6% of patients, followed by deferiprone (15.1%). The distribution of other chelation regimens was as follows: subcutaneous deferoxamine in 9.4% of patients, sequential deferoxamine plus deferiprone in 9.4%, combined deferoxamine plus deferiprone in 5.7%, and combined deferasirox plus deferiprone in 3.8%.

During longitudinal follow-up, dose adjustments were observed; however, switches to a different chelator class were infrequent (<10%).

## 4. Discussion

This study investigated for the first time longitudinal changes in LV T1 values over 18 months in patients with TDT.

Examination of baseline characteristics indicates that our cohort represents a well-managed population, with mean LV T2* values largely within the normal range, reflecting a low prevalence of overt MIO. This is likely because most patients were not naïve to T2* CMR, and previous T2* assessments, performed as part of routine clinical care, had guided adjustments in iron chelation therapy, contributing to effective control and normalization of myocardial iron over time [14].

The moderate but significant correlation between LV T1 and T2* confirms their shared association with myocardial iron loading [16], while indicating that the two parameters are not interchangeable. Notably, more than one quarter of patients exhibited reduced T1 values despite normal T2*, confirming the ability of native T1 mapping in detecting early or subtle myocardial tissue alterations not captured by T2* and highlighting the complementary value of T1 mapping, particularly in well-treated populations with controlled cardiac iron [20,21]. Patients with reduced T1 despite normal T2* were significantly older than the rest of the cohort, while showing comparable ferritin levels, chelation regimen, and cardiac function, suggesting that age, and thus longer cumulative exposure to disease-related factors, may contribute to myocardial involvement not identified by conventional markers. Clinically, these findings support the integration of T1 mapping into standard CMR protocols to refine risk stratification and guide timely, individualized adjustments in chelation therapy, particularly in older patients where reassuring T2* values may not fully exclude myocardial alterations. While T2* relaxometry remains the validated gold standard for the direct quantification of myocardial iron, reduced native T1 mapping can act as an ‘early warning system’. Importantly, this should not be interpreted as evidence of superiority, but rather as an integrative role, considering that T1 is a more composite marker influenced by a broader range of pathophysiological factors.

Longitudinally, the mean LV T1 did not change significantly, indicating overall myocardial tissue stability at the population level. Nevertheless, individual trajectories varied substantially. A notable proportion of patients with initially normal T1 values developed reduced T1 at follow-up, while only about one quarter of those with reduced baseline T1 normalized over time. These findings indicate that cohort-level analyses may underestimate clinically meaningful patient-specific changes and highlight the importance of longitudinal, individualized assessment rather than reliance on group averages alone. Serial assessment of T1 may provide additional clinical information when myocardial tissue changes occur in the presence of stable or normal T2* values. In particular, it may help identify divergence between myocardial tissue evolution and iron burden over time, especially in patients with previously normal or borderline-normal T2*. Importantly, normalization of T1 was consistently accompanied by normal T2* values, supporting the interpretation that improvements in T1 reflect reduced myocardial iron. The significant correlation between changes in LV T1 and T2* over time confirms that dynamic variations in myocardial iron burden influence both parameters in parallel, even though baseline T2* did not predict subsequent T1 changes.

Although the increase in LV T2* was relatively small, it was statistically significant, confirming its value in monitoring dynamic changes in myocardial iron. The limited magnitude of change likely reflects the overall stability of the cohort, in which chelation therapy was generally well controlled with infrequent modifications during follow-up, resulting in relatively stable myocardial iron status over time. The significant increase observed in T2*, but not in T1, can be explained by several factors. First, the relationship between relaxation times and iron concentration is nonlinear: T1 decreases with increasing iron less steeply than T2* [16], so once iron is present, small fluctuations in myocardial iron may produce minimal measurable T1 changes. Second, T1 is not specific for iron and is influenced by chronic tissue alterations such as diffuse fibrosis, which increases T1 [28,29]. Previous studies have shown that myocardial extracellular volume (ECV), a validated surrogate for diffuse fibrosis [30,31,32], is significantly increased and associated with MIO in thalassemia [33,34]. When limited iron overload and fibrosis coexist, their opposing effects can balance each other, yielding T1 values within the normal range. Diffuse fibrosis is at least partially reversible [35,36], and its gradual regression, facilitated by reduction of myocardial iron, likely contributes to the longitudinal stability of T1 even when T2* improves. T1 can be shortened by free radicals generated during oxidative stress [37]. In thalassemia, iron overload is a major, but not the sole, contributor to oxidative stress [38,39]; therefore, even after iron removal, a residual oxidative injury may persist and limit improvement in T1 values. Finally, it should also be taken into account that most of our patients had T2* values within the normal range and that the precision of T2* measurements decreases at higher values [15]. Consequently, small improvements in T2* at high baseline values may not fully reflect underlying tissue changes.

The inverse relationship between changes in LV T1 and baseline T1 values indicates a regression-to-the-mean effect and suggests that patients with more abnormal myocardial tissue characteristics at baseline may derive greater benefit from ongoing management. The lack of association between T1 changes and age, hemoglobin, or ferritin emphasizes the limited value of systemic markers in predicting myocardial tissue evolution and reinforces the role of CMR as the reference standard for cardiac assessment and longitudinal monitoring in TDT. We did not detect any significant correlation between changes in LV T1 or T2* and LV ejection fraction, likely because most patients exhibited normal or only mildly abnormal baseline values, which limited the range of detectable functional variation. Accordingly, LV ejection fraction remained stable over 18 months, indicating preserved systolic function in the setting of normal or near-normal myocardial iron burden.

### Limitations

This study has several limitations. The retrospective design may introduce selection bias, while the relatively small and selected sample, characterized primarily by low myocardial iron burden, may limit the generalizability of the findings and the detection of subtle but clinically meaningful changes. The single-center nature further limits the broader applicability of the results. Unlike T2*, which has been calibrated against histological measurements [40], T1 mapping currently lacks standardized validation against tissue samples, limiting direct quantification of myocardial iron. Finally, the relatively short follow-up duration restricts the assessment of mid- to long-term dynamics in myocardial tissue characteristics.

## 5. Conclusions

Our findings support native T1 mapping as a clinically valuable, complementary, though not interchangeable, tool to T2* for the assessment and longitudinal monitoring of myocardial iron overload in TDT. The combined use of T1 and T2* enhances the detection of subtle myocardial iron deposition, enables a more comprehensive evaluation over time, and may support more individualized chelation strategies. Further studies are needed to clarify whether routine integration of T1 mapping into CMR protocols can improve risk stratification and clinical management of cardiac complications in thalassemia.

## Data Availability

The data underlying this article cannot be shared publicly due to privacy reasons. The data will be shared on a reasonable request to the corresponding author.

## Abbreviations

TDT, transfusion-dependent thalassemia; CMR, cardiovascular magnetic resonance; MIO, myocardial iron overload; EMIOT, Extension-Myocardial Iron Overload in Thalassemia; MRI, magnetic resonance imaging; LV, left ventricular; SD, standard deviation.
